# Supplementary figures and images for: Exportin-mediated nucleocytoplasmic transport maintains Pch2 homeostasis during meiosis
Source: PLoS Genet. 2023 Nov 10;19(11):e1011026. doi: 10.1371/journal.pgen.1011026 (PMC10688877; doi:10.1371/journal.pgen.1011026)

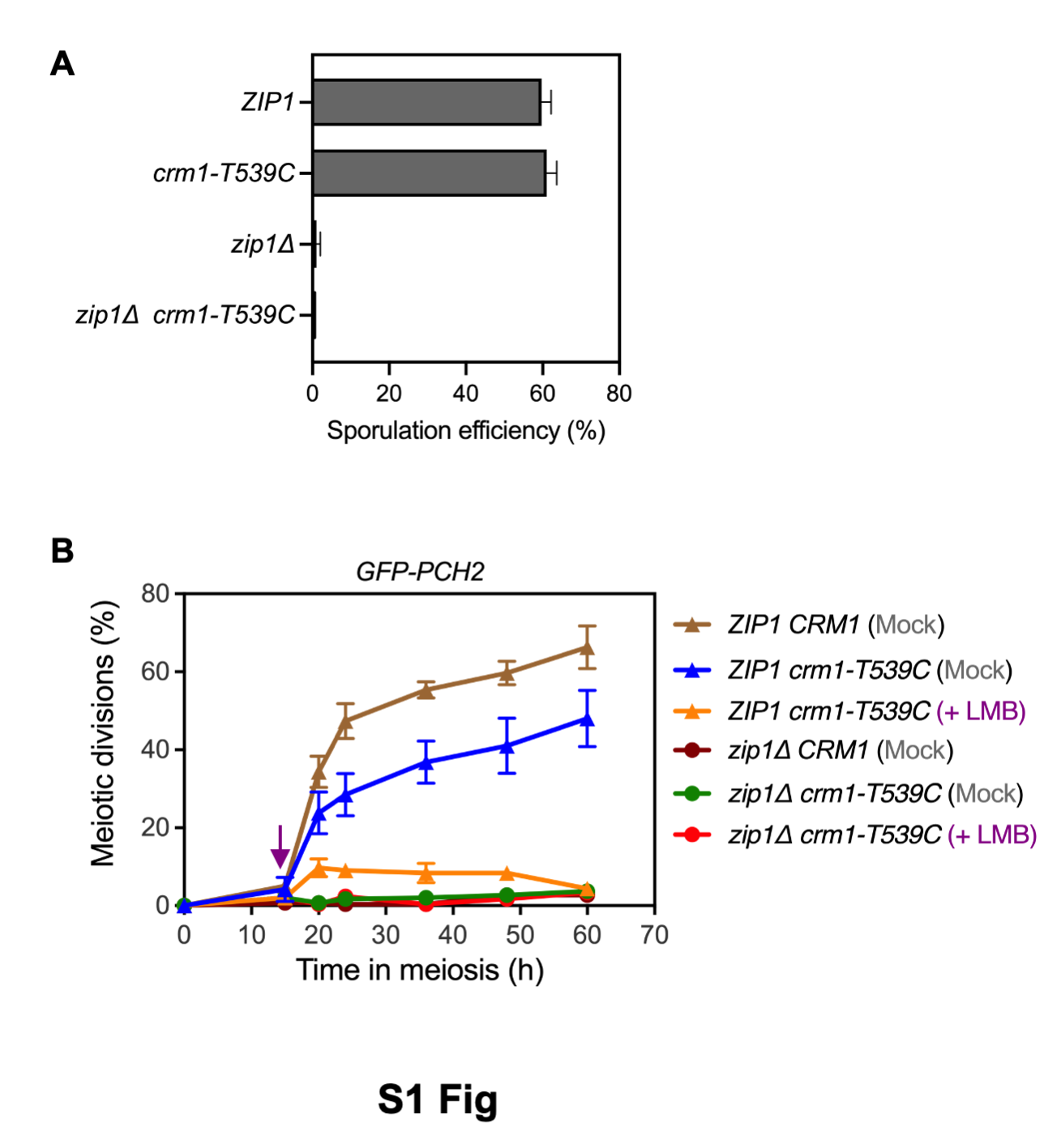

Supplement: S1 Fig — (A) Sporulation efficiency was examined after 3 days on sporulation plates. Error bars, SD; n = 3. At least 300 cells were counted for each strain. Strains are: DP421 (wild type), DP1717 (crm1-T539C GFP-PCH2), DP422 (zip1Δ), and DP1721 (zip1Δ crm1-T539C GFP-PCH2). (B) Time course analysis of meiotic nuclear divisions. The percentage of cells containing two or more nuclei is represented. Ethanol (Mock) or Leptomycin B (LMB) were added 15 h after meiotic induction (arrow). Error bars: SD; n = 3. At least 300 cells were scored for each strain at every time point. Strains are: DP1620 (ZIP1 CRM1 GFP-PCH2), DP1717 (ZIP1 crm1-T539C GFP-PCH2), DP1621 (zip1Δ CRM1 GFP-PCH2) and DP1721 (zip1Δ crm1-T539C GFP-PCH2). (TIF) [file pgen.1011026.s001.tif]

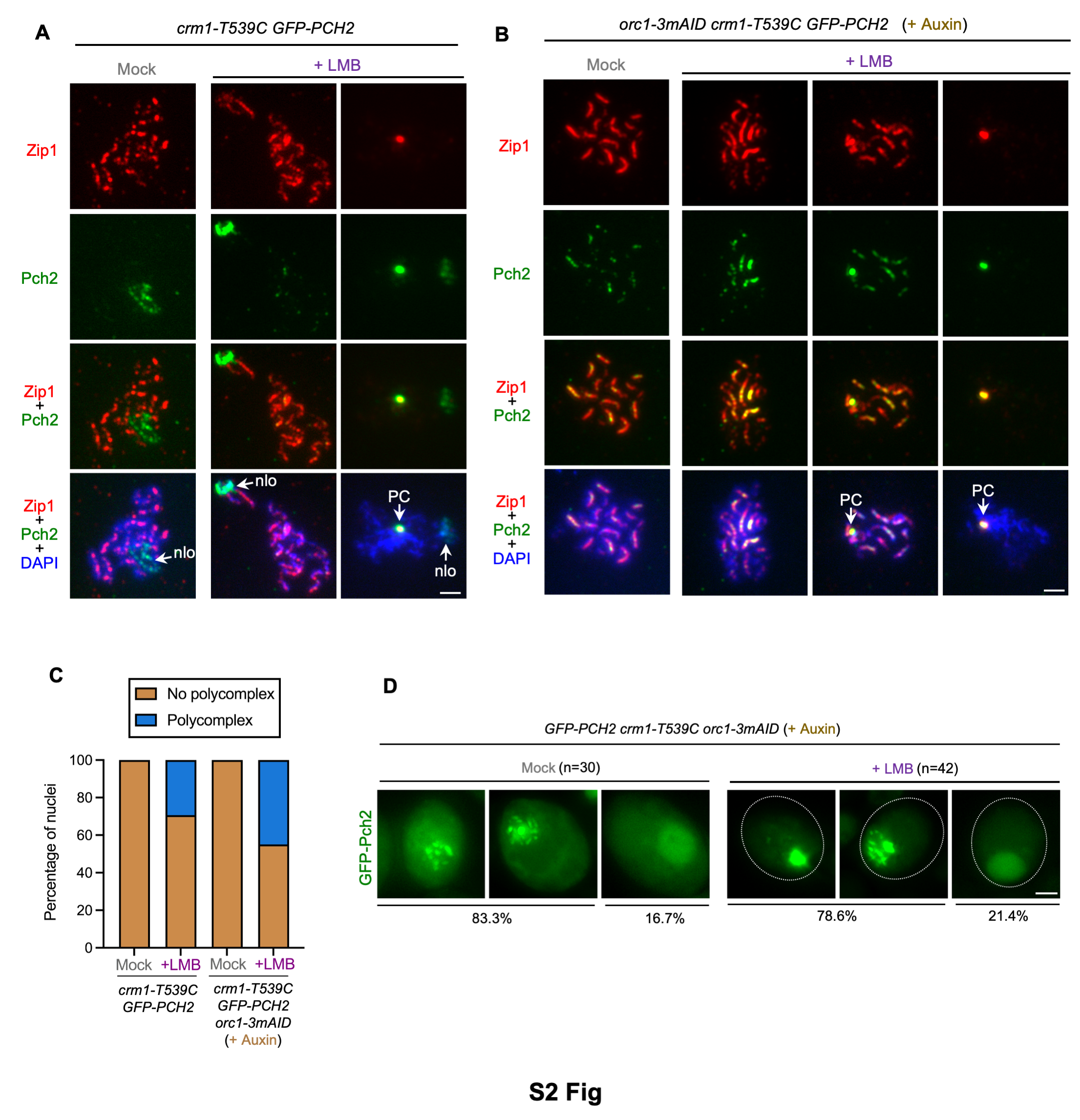

Supplement: S2 Fig — (A-B) Immunofluorescence of spread meiotic chromosomes at pachytene stained with anti-GFP antibodies (to detect GFP-Pch2; green), anti-Zip1 antibodies (red) and DAPI (blue). Representative nuclei are shown. In both, (A) and (B), cultures were mock-treated, or treated with 500 ng/ml LMB 15 h after meiotic induction. In (B), Auxin (500μM) was also added 12 h after meiotic induction to degrade Orc1. Spreads were prepared at 19 h. Arrows point to the rDNA region (nlo) and Polycomplex (PC). Scale bar, 2 μm. The strain in (A) is: DP1717 (crm1-T539C GFP-PCH2). The strain in (B) is: DP1885 (orc1-3mAID crm1-T539C GFP-PCH2). (C) Percentage of nuclei containing polycomplexes in the experiments shown in (A and B). Between 10 to 20 nuclei were counted for each strain and condition. (D) Quantification of the different patterns of Pch2 localization in the experiment presented in Fig 3A and 3B. Representative cells are shown. (TIF) [file pgen.1011026.s002.tif]

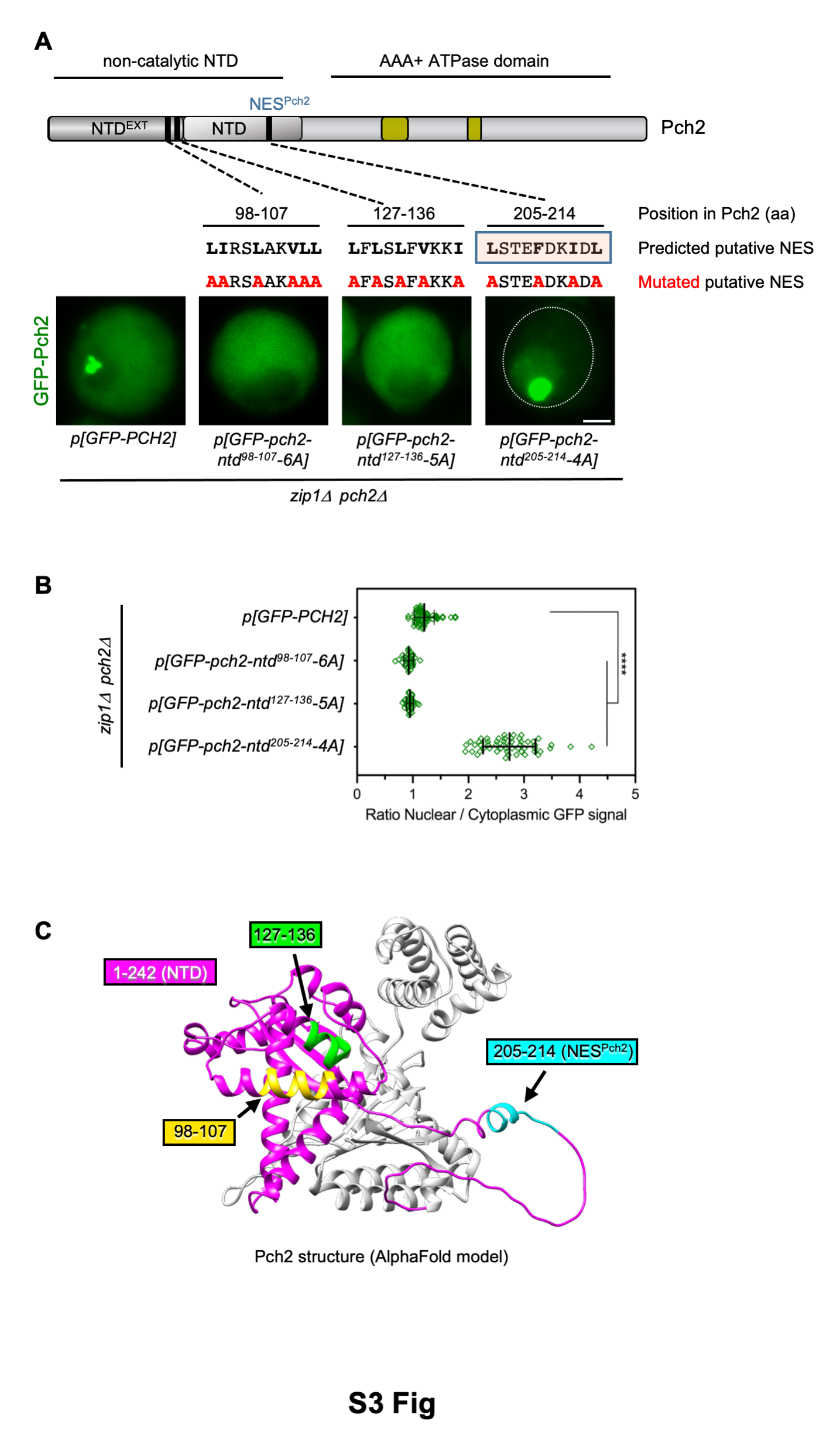

Supplement: S3 Fig — (A) Schematic representation of the S. cerevisiae Pch2 protein. The position and sequence of the three putative NESs predicted by LocNES in the non-catalytic N-terminal domain of Pch2 are depicted, as well as the corresponding mutants generated. The images show representative zip1Δ pch2Δ cells transformed with centromeric plasmids expressing wild-type GFP-PCH2 or the different mutated versions of the predicted NESs, as indicated. Note that only the mutation of the 205–214 region (boxed) leads to Pch2 accumulation in the nucleus. Images were taken 15 h after meiotic induction. The strain is DP1405 (zip1Δ pch2Δ) transformed with the centromeric plasmids pSS393 (GFP-PCH2), pSS448 (GFP-pch2-ntd98-107-6A), pSS451 (GFP-pch2-ntd127-136-5A) and pSS459 (GFP- pch2-ntd205-214-4A). (B) Quantification of the ratio of nuclear (including nucleolar) to cytoplasmic GFP fluorescent signal for the experiment shown in (A). Error bars, SD. (C) AlphaFold model of Pch2 structure. The N-terminal domain of Pch2 is labeled in pink. The positions of the putative NESs analyzed are labeled in yellow (98–107 region), green (127–136) and blue (205–214). (TIF) [file pgen.1011026.s003.tif]

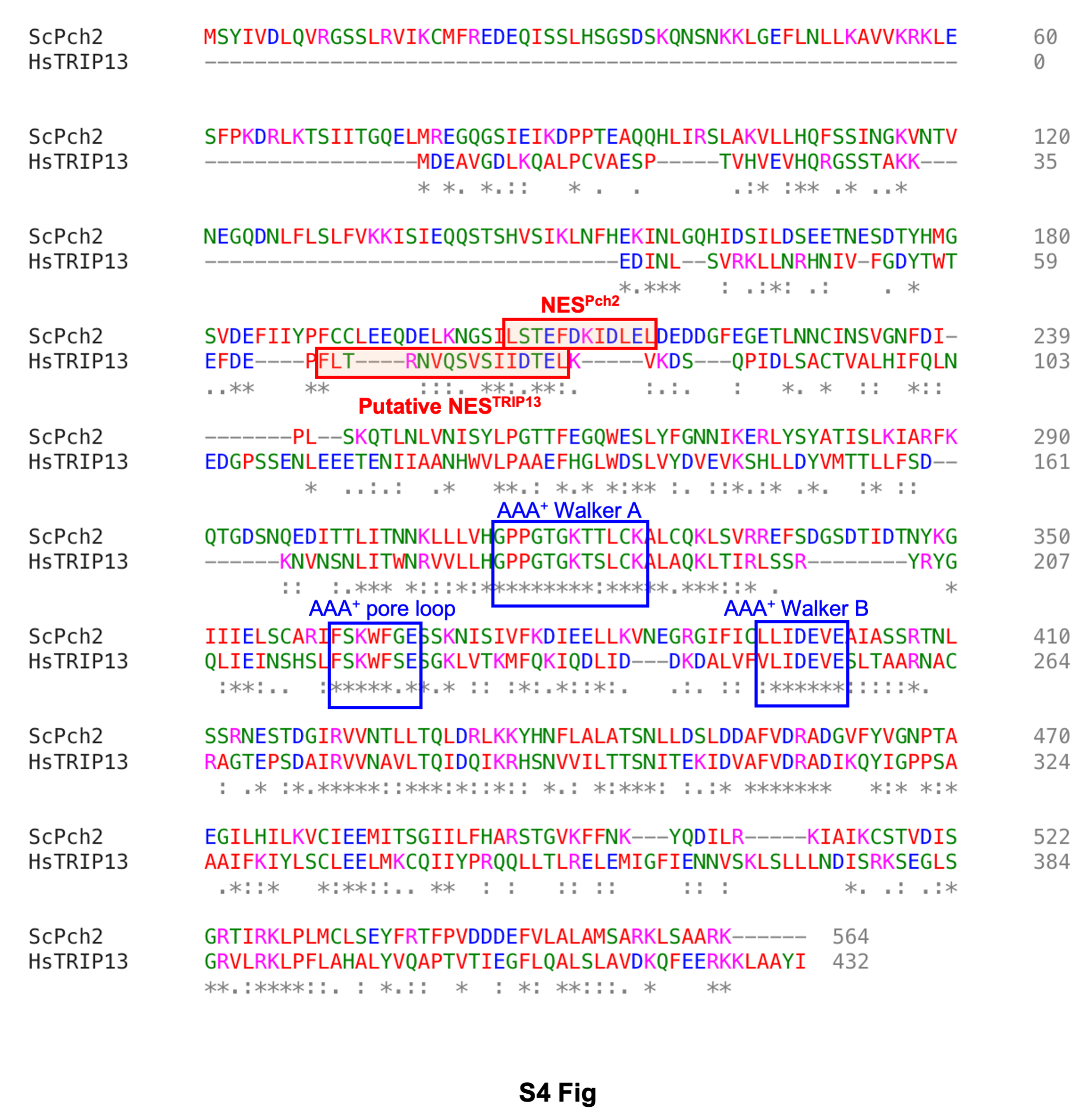

Supplement: S4 Fig — ClustalW alignment of the protein sequences of Pch2 orthologs from S. cerevisiae (ScPch2) and human (HsTRIP13). The characteristic AAA+ ATPase features are boxed in blue. The presumed NESs analyzed are boxed in red. The color code for amino acids is the following: AVFPMILW (small + hydrophobic -Y): red. DE (acidic): blue. RK (basic -H): magenta. STYHCNGQ (hydroxyl +sulfhydryl + amine + G): green. Alignment was performed at https://www.ebi.ac.uk/Tools/msa/clustalo/. (TIF) [file pgen.1011026.s004.tif]
