# Supplementary material for: Exportin-mediated nucleocytoplasmic transport maintains Pch2 homeostasis during meiosis
Source: PLoS Genet. 2023 Nov 10;19(11):e1011026. doi: 10.1371/journal.pgen.1011026 (PMC10688877; doi:10.1371/journal.pgen.1011026)
Supplement: S5 Fig — Sequence used for the AlphaFold prediction of NESTRIP13-Pch2-nes4A structure shown in Fig 6H. Relevant motifs are indicated. Pch2 N-terminal domain (NTD) is in pink color. (PDF) [file pgen.1011026.s005.pdf]

NES^{TRIP13}-Pch2-nes4A

FLTRNVQSVSIIDTELGAGAGAGAGASYIVDLQVRGSSLRVIKCMFREDEQISSLHSGSDSKQNSNKKLGEFLN
LLKAVVKRKLESFPKDRLKTSIITGQELMREGQGSIEIKDPPTEAQQHLIRSLAKVLLHQFSSINGKVNTVNEG
QDNLFLSLFVKKISIEQQSTSHVSIKLNFHEKINLGQHIDSILDSEETNESDTYHMGSVDEFIIYPFCCLEEQD
ELKNGSI**ASTEADKADA**ELDEDDGFEGETLNNCINSVGNFDIPLSKQTLNLVNISYLPGTTFEGQWESLYFGNN
IKERLYSYATISLKIARFKQTGDSNQEDITTLITNNKLLLVHGPPGTGKTTLCKALCQKLSVRREFSDGSDTID
TNYKGIIIELSCARIFSKWFGESSKNISIVFKDIEELLKVNEGRGIFICLLIDEVEAIASSRTNLSSRNESTDG
IRVVNTLLTQLDRLKKYHNFLALATSNLLDSLDDAFVDRADGVFYVGNPTAEGILHILKVCIEEMITSGIILFH
ARSTGVKFFNKYQDILRKIAIKCSTVDISGRTIRKLPLMCLSEYFRTFPVDDDEFVLALAMSARKLSAARK*

FLTRNVQSVSIIDTEL: 1-16 NES^{TRIP13}

GAGAGAGAGA: 17-26 flexible linker

ASTEADKADA: 230-239 nes^{Pch2}-4A

S5 Fig
