# Supplementary material for: Exportin-mediated nucleocytoplasmic transport maintains Pch2 homeostasis during meiosis
Source: PLoS Genet. 2023 Nov 10;19(11):e1011026. doi: 10.1371/journal.pgen.1011026 (PMC10688877; doi:10.1371/journal.pgen.1011026)
Supplement: S1 Table — (PDF) [file pgen.1011026.s006.pdf]

S1 Table. ***Saccharomyces cerevisiae*** **strains**

**Strain**	**Genotype***	**Source**
BR1919-2N	*MAT*a/*MAT*α *leu2-3,112 his4-260 thr1-4 trp1-289 ura3-1 ade2-1*	Roeder Lab
DP421	BR1919-2N *lys2ΔNheI*	PSS Lab
DP422	DP421 *zip1Δ::LYS2*	PSS Lab
DP881	DP421 *zip1Δ::LYS2 pch2Δ::TRP1 ndt80Δ::LEU2*	PSS Lab
DP1023	DP421 *pch2Δ::TRP1*	PSS Lab
DP1029	DP421 *zip1Δ::LYS2 pch2Δ::TRP1*	PSS Lab
DP1405	DP421 *zip1Δ::LEU2 pch2Δ::URA3*	PSS Lab
DP1620	BR1919-2N P_{HOP1}*-GFP-PCH2*	PSS Lab
DP1621	BR1919-2N *zip1Δ::LEU2* P_{HOP1}*-GFP-PCH2*	PSS Lab
DP1624	BR1919-2N P_{HOP1}*-GFP-PCH2/pch2Δ::TRP1*	PSS Lab
DP1625	BR1919-2N *zip1Δ::LEU2* P_{HOP1}*-GFP-PCH2/pch2Δ::TRP1*	PSS Lab
DP1639	BR1919-2N P_{HOP1}*-GFP-PCH2 ndt80Δ::kanMX3*	PSS Lab
DP1655	BR1919-2N *zip1Δ::LEU2* P_{HOP1}*-GFP-PCH2/pch2Δ::TRP1 ndt80Δ::kanMX3 LYS2/lys2ΔNheI*	PSS Lab
DP1717	DP421 *crm1Δ::hphMX4 pSS416(LEU2)-crm1-T539C* P_{HOP1}*-GFP-PCH2*	This work
DP1721	DP421 *zip1Δ::LYS2 crm1Δ::hphMX4 pSS416(LEU2)-crm1-T539C* P_{HOP1}*-GFP-PCH2*	This work
DP1787	DP421 *spo11-3HA-6His::kanMX4 pch2Δ::TRP1*	PSS Lab
DP1788	DP421 *spo11-3HA-6His::kanMX4* P_{HOP1}*-GFP-PCH2*	This work
DP1837	DP421 *zip1Δ::LYS2 crm1Δ::hphMX4 pSS416(LEU2)-crm1-T539C ndt80Δ::kanMX6* P_{HOP1}*-GFP-PCH2*	This work
DP1885	DP421 *orc1-3mAID::hphNT* P_{HOP1}*-OsTIR1::URA3 crm1Δ::natMX4 pSS416(LEU2)-crm1-T539C* P_{HOP1}*-GFP-PCH2*	This work
DP1886	DP421 *zip1Δ::LYS2 orc1-3mAID::hphNT* P_{HOP1}*-OsTIR1::URA3 crm1Δ::natMX4 pSS416(LEU2)-crm1-T539C* P_{HOP1}*-GFP-PCH2*	This work
DP1927	DP421 *crm1Δ::hphMX4 pSS416(LEU2)-crm1-T539C ndt80Δ::kanMX6* P_{HOP1}*-GFP-PCH2*	This work
DP1986	DP421 *zip1Δ::LYS2* P_{HOP1}*-GFP-pch2-nes4A/pch2Δ::TRP1*	This work
DP1988	DP421 *zip1Δ::LYS2* P_{HOP1}*-GFP-pch2-nes4A/pch2Δ::TRP1 ndt80::LEU2*	This work
DP1992	DP421 *zip1Δ::LYS2* P_{HOP1}*-GFP-*NES^{PKI}*-pch2-nes4A/pch2Δ::TRP1*	This work
DP2003	DP421 *zip1Δ::LYS2* P_{HOP1}*-GFP-*NES^{PKI}*-pch2-nes4A/pch2Δ::TRP1 ndt80Δ::kanMX6*	This work

DP2025	DP421 *zip1Δ::LYS2* P_{HOP1}*-GFP-NES*^{TRIP13}*-pch2-nes4A/pch2Δ::TRP1*	This work
DP2033	DP421 *zip1Δ::LYS2* P_{HOP1}*-GFP-nes7A*^{TRIP13}*-pch2-nes4A/pch2Δ::TRP1*	This work
DP2046	DP421 *spo11-3HA-6His::kanMX4* P_{HOP1}*-GFP-pch2-nes4A*	This work
DP2052	DP421 P_{HOP1}*-GFP-pch2-nes4A*	This work
DP2053	DP421 P_{HOP1}*-GFP-pch2-nes4A ndt80Δ::kanMX6*	This work

*All strains are diploids isogenic to BR1919 and, unless specified, homozygous for the indicated markers. DP421 is a *lys2* version of the original BR1919-2N.
