# Supplementary material for: Exportin-mediated nucleocytoplasmic transport maintains Pch2 homeostasis during meiosis
Source: PLoS Genet. 2023 Nov 10;19(11):e1011026. doi: 10.1371/journal.pgen.1011026 (PMC10688877; doi:10.1371/journal.pgen.1011026)
Supplement: S2 Table — (PDF) [file pgen.1011026.s007.pdf]

S2 Table. Plasmids

Plasmid name	Vector	Relevant description	Source / Reference
pSS393	pRS314	*TRIP1 CEN6* P_{HOP1}*-GFP-PCH2*	[1]
pSS416	pRS315	*LEU2 CEN6 crm1-T539C*	[2]
pSS448	pRS314	*TRIP1 CEN6* P_{HOP1}*-GFP-pch2-ntd*$^{98\text{-}107}$*-6A*	This work
pSS451	pRS314	*TRIP1 CEN6* P_{HOP1}*-GFP-pch2-ntd*$^{127\text{-}136}$*-5A*	This work
pSS459	pRS314	*TRIP1 CEN6* P_{HOP1}*-GFP-pch2-nes4A*	This work
pSS462	pRS314	*TRIP1 CEN6* P_{HOP1}*-GFP-NES*^{PKI}*-pch2-nes4A*	This work
pSS472	pRS314	*TRIP1 CEN6* P_{HOP1}*-GFP-NES*^{TRIP13}*-pch2-nes4A*	This work
pSS474	pRS314	*TRIP1 CEN6* P_{HOP1}*-GFP-nes7A*^{TRIP13}*-pch2-nes4A*	This work

References

[1] Herruzo E, Santos B, Freire R, Carballo JA, San-Segundo PA. Characterization of Pch2 localization determinants reveals a nucleolar-independent role in the meiotic recombination checkpoint. Chromosoma. 2019;128(3):297-316. doi: 10.1007/s00412-019-00696-7

[2] Moriggi G, Nieto B, Dosil M. Rrp12 and the Exportin Crm1 participate in late assembly events in the nucleolus during 40S ribosomal subunit biogenesis. PLoS Genet. 2014;10(12):e1004836. doi: 10.1371/journal.pgen.1004836
