# Supplementary material for: Exportin-mediated nucleocytoplasmic transport maintains Pch2 homeostasis during meiosis
Source: PLoS Genet. 2023 Nov 10;19(11):e1011026. doi: 10.1371/journal.pgen.1011026 (PMC10688877; doi:10.1371/journal.pgen.1011026)
Supplement: S3 Table — (PDF) [file pgen.1011026.s008.pdf]

S3 Table. Antibodies

Antibody	Host and type	Application* (Dilution)	Source / Reference
Hop1	Rabbit polyclonal	IF (1:300)	(1)
Hop1-T318-P	Rabbit polyclonal	WB (1:1000)	(2)
Pgk1 (22C5D8)	Mouse monoclonal	WB (1:5000)	Molecular Probes 459250
Pch2	Rabbit polyclonal	WB (1:2000)<br>IF (1:200)	(3)
Nsr1 (31C4)	Mouse monoclonal	IF (1:200)	ThermoFisher MA1-10030
Zip1	Rabbit polyclonal	IF (1:200)	(4)
GFP (JL-8)	Mouse monoclonal	IF (1:200)	Clontech 632381
Anti-mouse HRP	Sheep polyclonal	WB (1:5000)	GE Healthcare NXA931
Anti-rabbit HRP	Donkey polyclonal	WB (1:5000)	GE Healthcare NA934
Anti-rabbit Alexa Fluor 594	Goat polyclonal	IF (1:200)	Molecular Probes A11012
Anti-rabbit Alexa Fluor 488	Goat polyclonal	IF (1:200)	Molecular Probes A11034
Anti-mouse Alexa Fluor 594	Goat polyclonal	IF (1:200)	Molecular Probes A11032
Anti-mouse Alexa Fluor 488	Goat polyclonal	IF (1:200)	Molecular Probes A11029

*WB, western blot; IF, immunofluorescence

1. Smith, A.V. and Roeder, G.S. (1997) The yeast Red1 protein localizes to the cores of meiotic chromosomes. *J Cell Biol*, **136**: 957-967.

2. Penedos A, Johnson AL, Strong E, Goldman AS, Carballo JA, Cha RS (2015) Essential and Checkpoint Functions of Budding Yeast ATM and ATR during Meiotic Prophase Are Facilitated by Differential Phosphorylation of a Meiotic Adaptor Protein, Hop1. *PLoS ONE* **10**: e0134297.

3. Herruzo, E., Santos, B., Freire, R., Carballo, J.A. and San-Segundo, P.A. (2019) Characterization of Pch2 localization determinants reveals a nucleolar-independent role in the meiotic recombination checkpoint. *Chromosoma*, **128**: 297-316.

4. Sym M, Engebrecht JA, Roeder GS (1993) ZIP1 is a synaptonemal complex protein required for meiotic chromosome synapsis. Cell 72:365–378
