# Supplementary material for: Exportin-mediated nucleocytoplasmic transport maintains Pch2 homeostasis during meiosis
Source: PLoS Genet. 2023 Nov 10;19(11):e1011026. doi: 10.1371/journal.pgen.1011026 (PMC10688877; doi:10.1371/journal.pgen.1011026)
Supplement: Attachment — Submitted filename: Response to referees_PGENETICS-D-23-00808_2.pdf [file pgen.1011026.s012.pdf]

Response to comments from the Editor and Reviewers (manuscript PGENETICS-D-23-00808).

We are very grateful to the editor and all reviewers for their constructive comments, the thorough corrections, and the excellent suggestions to improve the manuscript.

In the responses below, lines refer to the "clean" version of the manuscript without the track-changes option activated. The version with the marked changes is also included in the submission.

Response to the Editor

1. Line 162. While normal sporulation efficiency is shown for crm1-T539C, meiotic kinetics is not. Please include a CRM1 control so that the timing of nuclear divisions can be compared.

We now show the meiotic kinetics of the *CRM1* control for comparison (S1B Fig), and we have included the corresponding explanation in the text (lines 168-171).

2. Line 176-179. Since the current work does not include a nucleolar marker for this experiment, this sentence needs to be reworded to indicate that this identification was made previously. Suggest "in mock-treated wild-type ZIP1 cells, Pch2 localized to one side of the nucleus in a region that was previously shown to correspond to the nucleolus (reference),"and in following text (i.e. description of zip1Δ experiments) please also make it clear that this area is presumed to be the nucleolus because it was shown to be so in previous experiments, provide references, etc.

We have made the suggested changes including the appropriate references (lines 185-187 and 191-192)

3. Lines 222 and following. These experiments do not measure the meiotic recombination checkpoint—they examine the meiotic response to double strand breaks (i.e. DSB-provoked phosphorylation of Hop1 by DNA damage response kinases). It is not possible to measure actual checkpoint activity because of the confounding effects of LMB. Therefore, please reword to indicate that what is being detected is the DNA damage response, not the checkpoint itself. Perhaps you could refer to the later results in Figure 4H in support of the conclusion that Pch2 accumulation in the nucleus interferes with the checkpoint itself. Please note, however, that this is a complicated conclusion; reduced Hop1 levels on chromosomes in cells with increased nuclear Pch2 might also reduce DSB formation, which would also compromise the checkpoint. Unless it can be shown that DSB levels are unaffected, the conclusions should be softened

I think we are largely dealing here with a semantic or conceptual issue of what a cell-cycle checkpoint is. We consider that a checkpoint is a surveillance mechanism that monitors a biological process (in this case the presence of unrepaired meiotic DSBs) and generates a signal. This signal is transduced through a signaling cascade (in this case involving sequential phosphorylation events by Mec1 and then Mek1) and results in different cellular responses, including, but not only, cell cycle arrest/delay. Other checkpoint outcomes, depending on the cell cycle type/stage and the original insult, could be regulation of DNA repair/recombination, stabilization of replication forks, regulation of dNTP pools, apoptosis (in higher

organisms), etc. We believe that this is the generally accepted notion in the field of DNA integrity checkpoints (mitotic or meiotic).

If one interprets the measurement of checkpoint activity exclusively as the measurement of meiotic cell cycle progression/arrest itself, of course, this is not possible to perform in conditions where the cell cycle is blocked by other means (i.e., LMB treatment or *ndt80Δ* mutation), as the editor remarks. However, we consider that, in these cases, checkpoint activity can be monitored at other levels in the response pathway by molecular markers and, indeed, Hop1-T318 phosphorylation has typically been used and it is regarded as a valid readout for checkpoint signaling because it reflects the activity of the upstream checkpoint kinase Mec1. Whether it is called "meiotic DNA damage response" or "meiotic recombination/prophase checkpoint response" could be just a terminology choice, because they both reflect basically the same general concept, and it does not affect the strength or the softness of the conclusions. For clarification, we have made a remark in this way according with the editor's comment (lines 236-237).

On the other hand, we do agree with the editor that the status of checkpoint activity when Pch2 localization is altered may be influenced by DSB levels; however, we note that the link Pch2-Hop1-DSBs is complex and not necessarily linear; for example, in a *pch2Δ* null mutant, there is more chromosomal Hop1, but DSBs are reduced (Farmer et al., PLOS One 2012). In any case, we have added a sentence expressing the possibility mentioned by the editor to broaden the interpretation (lines 247-250).

4. Making underlying data available only after the paper is accepted is not an option, because the underlying data need to be reviewed to make sure that they are provided in an appropriate format, etc. For example, underlying data for graphs that show ratios (i.e. nuclear/cytoplasmic) should be provided as original data, not as already-calculated ratios. So, please include the data underlying graphs in the next revision.

We now provide the underlying numerical data for all quantifications in S1 File. Raw data, as well as a statistics summary in S2 File.

Response to Reviewer#1

The manuscript by Herruzo provides compelling evidence that the cytoplasmic localization of Pch2 which the authors have previously published depends upon the active export of Pch2 from the nucleus using the Crm1 protein. Furthermore they have identified a nuclear export sequence in the amino terminus of Pch2, as well as its mammalian ortholog Trip13. By manipulating Pch2 localization through inhibition of Crm1 binding by LMB or deleting/adding nuclear export signal to the Pch2 N-terminus, they built on their previous work showing that when Pch2 accumulates in the nucleus, Hop1 is decreased on chromosomes and checkpoint activity is comprised. The experiments are well controlled and very rigorously done and, especially with the connection to Trip13, will be of interest to the meiosis community. My only comments are minor ones meant to correct some English language mistakes or improve the clarity of the presentation.

Minor comments

Line 39: "focalized" is not a word. Localized could be used instead.

We have made the change as suggested (line 39). However, although it may not be widely used, we note that the word "focalized" does exist with the meaning of "become confined to a limited area" (https://www.merriam-webster.com/dictionary/focalized#medicalDictionary=)

Line 49: traffic should be trafficking

Corrected (line 48).

Line 50: …involving nuclear export via…

Corrected (line 50).

Line 58-59…it would helpful if the words were written in the temporal order they occur in a yeast cell: pairing, recombination and synapsis.

We have made the change as suggested (line 77).

Line 65: define the LINC acronym

We have defined the acronym (line 83).

Line 70, the authors are correct that in the context of the SC the protein cores containing Hop, Red1 and Rec8 are referred to as lateral elements. But in the abstract and throughout the paper this term is never used again. Instead, Hop1 is referred to as an axial protein—which is also correct. To help a broader audience, explicitly say that lateral and axial refer to the same structures, just in the presence or absence of the central region.

We have included that statement according with the reviewer's suggestion (lines 90-91).

Line 105: two papers that should be cited for the fact that Hop1 interacts with Red1 are de los Santos and Hollingsworth, JBC 1999 and Bailis and Roeder Cell 2000.

We have included the references (line 118).

Line 204: only associated with the rRNA region…

Corrected (line 211).

Line 222: It would be helpful to a general audience to explain that in zip1Δ mutants the absence of the central region does not prevent crossovers from connecting the homologous axial elements. This explains why the unsynapsed homologs are connected in Figure 2A.

As suggested, we have now explained that unsynapsed axial elements are still connected in the *zip1Δ* mutant; however, we have included this explanation in the introduction (lines 91-92) to avoid distraction of the reader from the main point of the experiment shown in Fig 2A, which is the impaired localization of Hop1 upon LMB treatment independent of the presence of axial associations.

Line 226, In Figure 2A +LMB, the intensity of Hop1 staining is clearly less than without the drug. But the picture shown does not support the statement that the axial staining is less continuous.

We have removed the statement about the "continuity" of Hop1 staining (line 230).

Line 226, "To elude" is an incorrect use of this word. "To prevent" or "To circumvent" would be better.

Corrected (line 231).

Line 240: write out nuclear localization signal instead of using the acronym

We have defined the acronym (line 245).

Line 241: …arguing that simply blocking Pch2 nuclear export…

Corrected (line 246).

Line 284: don't use undefined acronyms in the title. Also, "drives" is too active a verb—it suggests the NES provides the motive force for nuclear export. Say instead, "A nuclear export sequence in the amino-terminus of Pch2 promotes export out of the nucleus".

Changed as suggested (lines 293-294).

Line 286, for more concise writing, avoid unnecessary words such as "It is well known…". Say instead, Crm1 exportin binds proteins possessing nuclear export signals…

Changed as suggested (line 296).

Line 324: delete "exquisite" as this is editorializing and subjective in the results

Corrected (line 341).

Line 343: delete "remarkably", again this is editorializing in the results.

Corrected (line 382).

Figure legend 1: for clarity, define the LMB acronym in panel A. What are the cytoplasmic tails hanging off the nuclear pore?

We have defined the LMB acronym in the legend of Fig. 1 (line 863). The cytoplasmic tails hanging out the nuclear pore represent the so-called "nuclear pore cytoplasmic filaments", which are filamentous extensions on the cytoplasmic side of the nuclear pore complex (NPC). The Nup159, Nup82 and Nup42 yeast nucleoporins contribute to this structure (please, see https://www.yeastgenome.org/go/GO:0044614). The "nuclear pore basket" present in the nuclear side, is also represented. However, since the work presented in the manuscript is not directly related to the NPC structure, we do not consider appropriate adding more detailed information about "irrelevant" (for this work) NPC parts that would result in a more crowded figure.

For Figure 5A, the label for the middle panel could be PCH2 + LMB or pch2-NES. This would incorporate the findings presented by the authors in the first part of the paper in their model.

We understand the reviewer's comment and, indeed, we had a debate about this issue while elaborating the figure for the first submission. We do agree in the sense that the results shown in the "first part" of the paper related to the effects of LMB are not incorporated in the model. However, those results showing that Pch2 follows the exporting pathway led to the "second part" where we identify and characterize the actual NES promoting Pch2 export and its direct impact on the checkpoint. Since LMB may have a broader effect and its action is already schematically represented in Fig 1A, we prefer to leave the model figure (now Fig 7) as it is, with a straightforward flow to convey the main message: the normal situation (left panel), the *pch2-nes4A* mutant (middle panel), and the rescue of this mutant by an ectopic NES (right panel). Nevertheless, we have slightly modified the figure, including also the fusion to the canonical ectopic NES from PKI in the right panel, which was not mentioned in the previous version. We have also added *zip1Δ* to the genotypes to make clear that the model refers to a checkpoint situation. The figure legend has been modified accordingly.

For Table S2, Change "Relevant parts" to Yeast genotype

That table contains the plasmid list. In my opinion, using the term "yeast genotype" in a plasmid description is misleading. We have changed "relevant parts" to "relevant description" in S2 Table.

Table S3, The authors should add the sources of the secondary antibodies they used and the dilutions, as well as the incubation conditions for both the primary and secondary antibodies.

We have added in S3 Table the secondary antibodies we used and the dilutions. The incubation conditions (time, temperature, blocking…) have been included in the materials and methods section (lines 507-510 and 518-521).

In the Bibiography, only the first word of the title and proper nouns should be capitalized.

We have corrected that issue arising from the Endnote formatting tool.

Response to Reviewer#2

The is a straightforward characterization of the regulation of Pch2's nuclear export in budding yeast and provides important information about the balance required for the nuclear import and export of PCH2 to regulate the recombination checkpoint and meiotic progression. The authors demonstrate that Pch2's nuclear export requires the conserved export factor Crm1, identify the nuclear export signal in Pch2, demonstrate that nuclear export is important to maintain the meiotic recombination checkpoint and show that the signal for export on Pch2/TRIP13 is conserved between budding yeast and mammals. This is a relevant manuscript for the meiosis field, provides important insight about the regulation of Pch2 and is a rigorous study. In particular, I appreciated the authors rigor in adding the PKI NES to verify that the mutation of Pch2's NES did not effect the protein's function. I have two major concerns about what I think are important controls and some minor concerns that should be addressed before publication to make the paper more accessible.

Major concerns:

What are the consequences of mutating Pch2's NES on meiosis in a strain with functional ZIP1? In particularly, what does spore viability, meiotic progression, and polycomplex formation look like? This might be be useful to determine whether the specific phenotypes observed when blocking all nuclear export (Figures S1 and S2) are because of defects in exporting other factors or defects because of exporting PCH2 specifically. Also, given that Pch2 ensures Hop1 availability for loading onto meiotic chromosomes, it may address whether enforced nuclear accumulation of Pch2 has consequences for normal meiotic progression.

We have carried out the analyses suggested by the reviewer in the *pch2-nes4A* mutant (*ZIP1* background). The results are presented in the new Fig 5, and they are described and discussed in lines 347-366. These findings revealed that *pch2-nes4A* has little phenotypic impact on *ZIP1* strains. This was the expected result because we have previously shown that even more massive nuclear accumulation of Pch2 forced by other means, such as the fusion to a strong NLS (NLS^{SV40}-Pch2), provokes no significant effects on unperturbed *ZIP1* meiosis (Herruzo et al. PLOS Genetics, 2021). Moreover, it is also known that meiotic progression and spore viability are also minimally altered in the *pch2Δ* single mutant, suggesting that Pch2 function is less relevant in normal meiosis. Only in conditions where DSB levels are reduced (i.e., *spo11-3HA*), spore viability is compromised when Pch2 function/localization is altered (Martini *et al*, Cell, 2006; Zanders & Alani, PLOS Genetics, 2009; Herruzo *et al.*, PLOS Genetics 2021). In addition, we did not observe polycomplex assembly in the *pch2-nes4A* single mutant, although it was increased in *ndt80Δ pch2-nes4A*, implying that cell cycle arrest contributes to formation of these assemblies. Therefore, like *pch2Δ* and *NLS^{SV40}-pch2*, *pch2-nes4A* has a stronger effect in the *zip1Δ*-induced checkpoint response than in *ZIP1* meiotic progression.

What does Hop1 loading look like in zip1Δ GFP-pch2-nes4A ndt80 mutants? Does this correlate with the inability of this mutant to fully restore meiotic progression in zip1Δ? If not, this may also support the possibility that enforced nuclear accumulation of Pch2 has consequences for normal meiotic progression.

As suggested by the reviewer, we have analyzed Hop1 chromosomal distribution in *zip1Δ GFP-pch2-nes4A ndt80.* The results are shown in new panels of Fig 4 (now Fig 4D and 4E), and described in lines 326-329 and 337. This analysis revealed that Hop1 localization is impaired in this mutant although not to the same extent as in *zip1Δ pch2Δ*. Thus, Hop1 localization correlates with the kinetics of meiotic progression.

Minor concerns:

The authors use a mutation in crm1 and drug treatment to abrogate nuclear export. Is there a reason they do not use a null mutation in crm1? This information may be useful with those unfamiliar with the details of this field.

As we mention in the text (line 436), the *CRM1* gene is essential, precluding the use of a *crm1Δ* null mutation to abrogate nuclear export. In addition, the use of LMB allows us to conditionally block nuclear export at the desired time, in this case meiotic prophase.

Can the authors outline the nucleus in figures where they are comparing nuclear and cytoplasmic localization of GFP-Pch2? This would be relevant for Figures 1B and C, 3A, and 4A.

During the process of generating the figures containing fluorescence images of whole cells, we have performed several trial tests with various combinations of none, nuclear and/or cytoplasmic outlines. We reached to the conclusion that the simpler the better to visually reflect the differences between nuclear and cytoplasmic signal (of course, what is most relevant is the quantification). We opted for drawing outlines to mark the cell contour exclusively in those cases where the cytoplasmic signal is so low that it cannot be distinguished (i.e., particular cells in Figs 3A, 4A, new 6B, S2D and S3A). In the case of the nucleus, we observed that, when it is outlined, instead of emphasizing the visible difference between nuclear and cytoplasmic signals, visualization of those differences is indeed somewhat masked in many cases. Therefore, contrary to the reviewer's suggestion we have opted for leaving the nuclei without outlining. Please, see the figure below, corresponding to the panels shown in Fig 3A, as an example justifying our decision. Especially in the right panels, the contrast between Pch2 nuclear/cytoplasmic signal is less clear when the nuclei are outlined. In addition, we consider that the images with the full outline are overloaded.

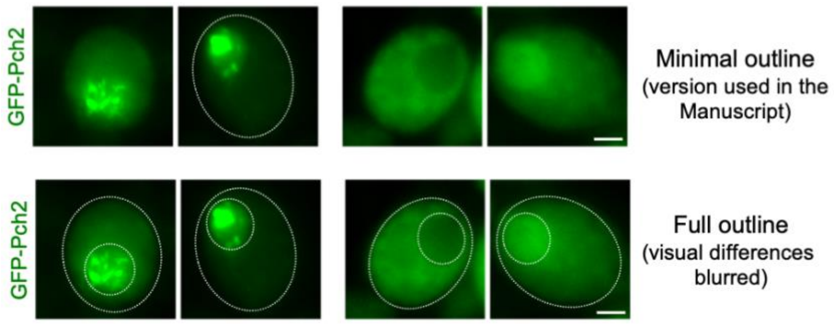

The authors mention several examples where they use ndt80 null mutants to avoid cell cycle timing as a possible confounder of their analysis. In examples where they are not using ndt80 mutants, can they mention in text the timepoints at which the spreads in 1F and G, 4A, C and D and their quantifications (Figures 1H-J, 4B and 4D) were performed? This information is present in the figure legends but I think it would also be useful in the text.

We have added that information in the text of the materials and methods section (lines 517-518). We consider that adding the information in the Results section is redundant with the Figure legends where, in fact, it is more contextualized together with the information relative to the time of LMB (Figs 1F and 1G legend), and auxin+LMB treatments (Figs 3C and 3D legend).

Is there chromosomal associated GFP-Pch2 in the mock treated zip1^ spreads (Figure 1G)? There appears to be Pch2 signals not at the nucleolus that are not present in the LMB rated zip1^ spreads.

Those particular dots are non-specific spurious foci that sometimes appear in IF spread preparations. These foci do not coincide with the DAPI-stained area and, therefore, are not associated with the chromosomes.

Line 201-202: "the accumulation of Pch2 in the nucleus was also associated to the increased formation of polycomplexes" should be "the accumulation of Pch2 in the nucleus was also associated with the increased formation of polycomplexes"

Corrected (line 208).

Line 204: "Pch2 is only associated to the rDNA region" should be "Pch2 is only associated with the rDNA region"

Corrected (line 211).

Line 241: "arguing that the simply block of Pch2 nuclear export by itself may account for the impaired checkpoint activity observed in LMB-treated zip1Δ cells." should be "arguing that simply blocking Pch2 nuclear export by itself may account for the impaired checkpoint activity observed in LMB-treated zip1Δ cells."

Corrected (line 246).

Line 304: italicize GFP-pch2-ntd205-214-4A or capitalize GFP-Pch2-ntd205-214-4A to indicate the protein

Corrected (line 312).

Response to Reviewer#3

Successful meiosis depends on the proper control of programmed DNA double strand break formation, which depends largely on the proper localization of the meiotic regulator Hop1. Hop1's localization to, and eventual removal from, meiotic chromosomes relies on the AAA+ ATPase Pch2 (TRIP13 in mammals), which remodels Hop1 and promotes its dynamic relocalization at different stages of meiotic prophase. In earlier work, the San-Segundo group has demonstrated that Pch2's localization in both the cytoplasm and the nucleus is important for its functions. In the current manuscript "Exportin-mediated nucleocytoplasmic transport maintains Pch2 homeostasis during meiosis," Herruzo et al. identify a nuclear export signal (NES) in Pch2 and show that nuclear export is important for its roles in Hop1 regulation (and meiotic checkpoint function). The work is well done, the conclusions are clear, and I generally support publication after the authors address a few outstanding issues (detailed below).

Major concerns:
I'm not sure I agree with the conclusions in the section "Pch2 nucleocytoplasmic traffic is independent of Zip1 and Orc1." The data in this section are clear, and the experiments are important. But the presence of Zip1 protein clearly does have an effect on the overall nuclear/cytoplasmic ratio of Pch2 - for example, compare the two purple datasets in Figure 3B, or in Figure 1D.

The reviewer is right, as we have previously reported (please, see Figs 3A, 3B or Fig 6B of Herruzo et al., PLOS Genetics 2021), and it is also shown in Fig 1D of this manuscript, in the absence of Zip1, the cytoplasmic fraction of Pch2 increases.

Related to the above, I think it would be important to compare +/- auxin data for the experiments in Figure 3A-3B. This would likely show that the presence or absence of Orc1 protein also has an effect on the nuclear/cytoplasmic ratio of Pch2.

As we showed in the original work generating and characterizing the *orc1-3mAID* mutant (Herruzo et al., Chromosoma, 2019), this is not a perfect conditional allele because, even in the absence of auxin, Pch2 nucleolar localization is somewhat affected. Of course, to completely abolish Orc1 function, we use *orc1-3mAID* in the presence of auxin for a full protein depletion, but the appropriate control to compare with is the wild-type *ORC1*. In any case, we have already reported the requested analysis of Pch2 subcellular distribution in the presence/absence of Orc1 (please, see Figs 1C and D of Herruzo et al., PLOS Genetics 2021). As envisioned by the reviewer, the nuclear/cytoplasmic ratio of Pch2 is altered, with increased cytoplasmic localization in cells lacking Orc1.

Thus, the difference between the authors' interpretation of these data and my own is one of emphasis: while the authors are correct that Pch2's transport per se does not involve Zip1 or Orc1, these proteins clearly do tend to retain Pch2 in the nucleus and therefore affect the intracellular distribution of Pch2.

We completely agree with the reviewer, this a matter of emphasis in the interpretation; both assertions are correct: 1) Pch2 nuclear transport per se does not involve Orc1 or Zip1, and 2) Orc1 and Zip1 affect the subcellular distribution of Pch2. Since this work is focused on Pch2 nucleocytoplasmic transport, we have

emphasized the first conclusion; nonetheless, we have added a final statement in this section reconciling both interpretations (lines 289-291).

Regarding the section describing NES identification, the authors should consider including supporting information from the AlphaFold structural model of Pch2. This model (available on the AlphaFold database web site) shows that residues 98-107 and 127-136 are in strongly-predicted alpha helices within the Pch2 N-terminal domain. Whereas, residues 205-214 are within a region that is predicted to be disordered in solution - perfect for an NES. This is nicely supportive and the authors may wish to mention it.

As suggested by the reviewer, we now include a new panel (S3C Fig) (described in lines 316-320) presenting the predicted AlphaFold structural model of Pch2 in which we have marked the three software-predicted sequences analyzed as putative NESs. As pointed out by the reviewer, only residues 205-214, encompassing the confirmed Pch2 NES in this work, are displayed in a disordered and highly accessible region for the interaction with Crm1. Two references supporting the notion that NESs are often located in disordered regions are also cited (lines 314-316).

I am very skeptical that the authors are testing what they think they are testing in the final Results section, where they transplant a putative NES from mammalian TRIP13 into Pch2 and observe rescue of some phenotypes. The structure of TRIP13 shows that this region (residues 65-80) is part of the structured TRIP13 N-terminal domain, and the hydrophobic residues in this region are largely buried in this domain. Thus, this sequence is highly unlikely to mediate nuclear export in the mammalian protein. When grafted onto Pch2, this stretch of sequence may well serve as a nuclear export sequence, simply because of the presence of solvent-exposed hydrophobic residues. This does not, however, prove the authors' assertion that the NES of Pch2/TRIP13 is evolutionarily conserved. I suggest either removing this section entirely or re-framing it significantly. Much of the "Concluding Remarks" would also have to be altered if this section were removed or re-framed.

In the new panels (F-H) of the new Fig 6, along with the predicted Pch2 model structure, we now show the location of the presumptive NES^{TRIP13} (residues 65-80) within the native TRIP13 structure, as well as within the AlphaFold-predicted structure of the NES^{TRIP13}-Pch2-nes4A fusion protein. As remarked by the reviewer, those residues are located in a structured region of TRIP13 (Fig 6G), whereas they are predicted to be in a highly-exposed unstructured zone when inserted at the beginning of Pch2-nes4A (Fig 6H). We do not fully agree, though, with the assertion that the putative NES^{TRIP13} is "largely buried" in the native TRIP13 NTD; it is on a side area of the NTD, and at the boundary of short unstructured stretches, which are features of some functional NESs (Lee et al., Sci Rep 2019). In any case, we do contemplate that the explanation suggested by the reviewer is feasible, and we have included that possible alternative interpretation for the rescue of *pch2-nes4A* by the putative NES^{TRIP13}.

Thus, according with the reviewer suggestion, we have significantly toned down the conclusions about the evolutionarily conservation of the NES in Pch2 and TRIP13 at different places in the text:

- We have changed the original title of the section "Evolutionary conservation of Pch2/TRIP13 nuclear export mechanism" to the more neutral "The checkpoint defects of *pch2-nes4A* are rescued by the fusion of a putative NES from human TRIP13" (lines 368-369).
- We have changed "these observations strongly suggest…" to "These observations are compatible with the notion that…" (lines 387-388).
- We have changed "contains…" to "may contain…" (line 389).
- We have added a description and interpretation of the predicted structures, and the alternative explanation indicated by the reviewer for the rescue of *pch2-nes4A* (lines 391-399), (Fig 6F-6H).
- We explicitly say that our results are not conclusive about the evolutionary conservation, and we have added a sentence indicating that additional studies in mammalian systems would be required to address this issue (lines 399-400).

In the concluding remarks section:
- We have changed "We provide evidence here for a possible…" to "It is tempting to speculate about the possibility of an …" (lines 410-411).
- We have changed "suggesting…" to "raising the possibility…" (line 416).
- We have changed "importance…" to "potential relevance…" (line 423).

In the abstract:
- We have removed "conserved…" (line 47).

In the title and legend of S4 Fig:
- We have removed "Conserved…" and changed the wording (lines 1043 and 1046).

Minor points:

Figure 2A - I think it would be helpful to have panels with each channel for this figure

We now show the individual channels for Pch2/Hop1 in the revised Fig 2A.

Figure 4B & 4G - color datasets in this panel consistent with the genotype coloring in other panels?

The consistent coloring pattern for other panels in Figs 1, 2 and 3 is grey symbols/lettering for mock-treated samples, and purple symbols/lettering for LMB treatments. Since the results shown in Figs 4B and 4G (now 6C) derive from different experimental approaches that do not involve LMB, a different color has been deliberately used to denote this difference.
